# Myeloid leukemia-derived galectin-1 downregulates CAR expression to hinder cytotoxicity of CAR T cells

**DOI:** 10.1186/s12967-023-04832-x

**Published:** 2024-01-06

**Authors:** Chuo Li, Shiyu Zuo, Lingling Shan, Huifang Huang, Haidong Cui, Xiaoming Feng

**Affiliations:** 1 State Key Laboratory of Experimental Hematology, National Clinical Research Center for Blood Diseases, Haihe Laboratory of Cell Ecosystem, Institute of Hematology & Blood Diseases Hospital, Chinese Academy of Medical Sciences & Peking Union Medical College, Tianjin, 300020 China; 2 Tianjin Institutes of Health Science, Tianjin, 301600 China; 3 Central Laboratory, Fujian Medical University Union Hospital, Fuzhou, 350001 China; 4 Department of Breast Surgery, The First Affiliated Hospital, School of Medicine, Zhejiang University, Hangzhou, 310000 China

**Keywords:** CAR down-regulation, Galectin-1, AML

## Abstract

**Background:**

Chimeric antigen receptor (CAR) T cells have shown significant activity in B-lineage malignancies. However, their efficacy in myeloid leukemia has not been successful due to unclear molecular mechanisms.

**Methods:**

We conducted in vitro and in vivo experiments to investigate whether myeloid leukemia cells directly induce CAR down-regulation. Furthermore, we designed a CD33 CAR^KR^ in which all lysines in the cytoplasmic domain of CAR were mutated to arginine and verified through in vitro experiments that it could reduce the down-regulation of surface CARs and enhance the killing ability. Transcriptome sequencing was performed on various AML and ALL cell lines and primary samples, and the galectin-1-specific inhibitory peptide (anginex) successfully rescued the killing defect and T-cell activation in in vitro assays.

**Results:**

CAR down-regulation induced by myeloid leukemia cells under conditions of low effector-to-tumor ratio, which in turn impairs the cytotoxicity of CAR T cells. In contrast, lysosomal degradation or actin polymerization inhibitors can effectively alleviate CAR down-regulation and restore CAR T cell-mediated anti-tumor functions. In addition, this study identified galectin-1 as a critical factor used by myeloid leukemia cells to induce CAR down-regulation, resulting in impaired T-cell activation.

**Conclusion:**

The discovery of the role of galectin-1 in cell surface CAR down-regulation provides important insights for developing strategies to restore anti-tumor functions.

**Supplementary Information:**

The online version contains supplementary material available at 10.1186/s12967-023-04832-x.

## Introduction

Acute myeloid leukemia (AML) accounts for almost a third of all leukemia diagnosed [1]. Chemotherapy has been the conventional treatment for AML over the past four decades. Still, approximately 40–45% of younger and less than one-third of adult patients achieve durable remission. Patients with relapsed or refractory AML (r/r AML) have worse outcomes, with overall survival (OS) estimated to be no more than 10% at three years [2–5]. The only curative treatment is allogeneic hematopoietic stem cell transplantation (HSCT). Still, it is an option for only a few patients [3]. Minimal residual disease (MRD) positive patients had significantly worse outcomes, which limits the application of HSCT, and relapse following HSCT is associated with poor prognosis [6, 7]. Therefore, finding new effective treatments for patients with r/r AML is an urgent clinical need.

Chimeric antigen receptor (CAR) T-cell therapy has effectively treated some certain B-cell malignancies, achieving high remission rates [7–9]. Thus, CAR-T cell therapy for AML may hold promise for the future. CAR-T cell therapies targeting CD123, CD33 and CLL-1 have shown preliminary efficacy and safety in clinical trials for r/r AML, though the low response rates for CAR-T cells in r/r AML indicate there are still challenges to overcome [10–13]. There are several barriers to developing effective CAR-T therapies for AML, such as antigen loss, poor T-cell persistence, and immunosuppressive microenvironments [14]. Potential solutions have been proposed to develop safe and effective AML CAR T-cell therapy. However, the efficacy still needs to be further improved, and the molecular mechanisms of r/r AML resistance to CAR-T cell therapy needs further study [15].

Some evidence suggests that relapse after CD19 CAR T-cell therapy is due to antigen-negative or antigen-low, which is attributed to trogocytosis between the CAR T cells and tumor cells [16, 17]. Some studies have also reported down-regulation of CAR after exposure to tumor antigens, but whether this phenomenon affects CAR-T cell efficacy is still controversial [16, 18–20]. In this study, we observed for the first time myeloid leukemia cells directly induce CAR down-regulation under the condition of a low effector: tumor ratio (E: T) in vitro and in vivo experiments, and this phenomenon was not observed in CAR T-cells in B-lineage tumor microenvironment under the same conditions. We also identified galectin-1 as a critical role in the down-regulation of cell-surface CAR, which impairs the anti-tumor effect of CAR-T cells.

## Materials and methods

### Cell lines

The cell lines, including Nalm6, Raji, SUP-B15, U937, THP-1, MOLM-13 and HL-60 were obtained from the Cell Resource Center of our institute and their identity was verified by short tandem repeat (STR) markers. Tumor cell lines overexpressing CD33, CLL-1, and CD123 antigens were constructed by lentiviral transduction, and they were further purified by cell sorting (FACS Aria II, BD Biosciences) or single-cell cloning. HEK293T cells and tumor cell lines were cultured as previously described [32]. T cells were cultured in T Cell Expansion Medium (Stem Cell Technologies), supplemented with 100 ng/ml recombinant human IL-2 (Peprotech). We obtained primary AML and ALL samples from the Department of Clinical Laboratory of the Institute of Hematology and Blood Diseases Hospital, CAMS. The study was conducted in accordance with the Declaration of Helsinki and was approved by the Ethics Committee of the Institute of Hematology, CAMS.

### Lentiviral production

One day before transfection, 1 × 10^7^ 293 T cells were inoculated in a T75 culture flask with DMEM complete medium. The 12 µg target vector plasmids were co-transfected into HEK293T with 2.5 µg Rev, 5 µg pMDL, and 3 µg VSVG helper packaging plasmid DNA using polyethyleneimine (PEI, Polysciences) transfection reagents. After 6 h of transfection, the medium was replaced with 10 ml of DMEM complete medium. Lentiviral supernatants were collected 48 h after transfection. They were then stored at – 80 ℃ and left to thaw at 4 ℃ before transduction.

### CAR constructs generation

CAR consisted of a single-chain fragment variable (scFv) specific to CD33 (clone My96) or CLL-1 (clone 1075.7) or CD123 (clone 32,716) fused to a CD8α hinge-transmembrane domain, a 4-1BB costimulatory and a CD3ζ signaling domains. All of the constructions have been expressed in the pCDH-EF1-MCS-Puro lentiviral vector.

### T cell isolation and production of CAR T cells

According to the product instructions, we selected human CD3^+^ T cells with the EasySepTM Human T Cell Enrichment Kit (Stem Cell Technologies). T cells were first activated using ImmunoCult™ Human CD3/CD28 T Cell Activator (Stem Cell Technologies), then CAR T cells were prepared by transducing lentivirus into activated T cells and cultured in T cell medium containing 100 ng/ml IL-2. The CAR T cells were used for the subsequent in vivo and in vitro experiments on the 10th or 11th day after activation if cell viability was greater than 90%.

### Flow cytometry and cell sorting

After staining for cell surface markers with monoclonal antibodies for 30 min at 4 ℃ in the dark, the cells were washed with 1 ml of 2% FBS in phosphate-buffered saline (2% FPB-PBS) and resuspended in 200 µl of 2% FBS-PBS. All samples were analyzed with an LSR Fortessa or CantoII (BD Bioscience), and data were analyzed using Flowjo software. Cell sorting was performed on FACS Aria II. Details of the antibodies were provided in the Additional file 1: Data S1.

### In vitro killing assay

Green fluorescent protein-positive (GFP^+^) tumor cells and purified CAR T cells or pCDH T cells were cocultured in 150 µL RPMI-1640 medium in 96-well round-bottomed plates (Corning, Lowell, USA) for 12–16 h. In the figure legends, different E: T ratios are indicated. After coculturing, we added precision count beads^™^ (BioLegend) to each sample to obtain absolute counts of cells by flow cytometry. For experimental details and calculating formula, we refer to the Precision Count Beads™ Protocol and Applications. The killing efficacy of CAR T cells against tumor cells was calculated using the following equation: CAR T cells killing efficacy = (live tumor cells in pCDH wells-live tumor cells in the sample wells)/live tumor cells in pCDH wells. We added NH_4_Cl (20 mM), galectin-1 specific inhibitor anginex (Jiangsu Jitai, 10 µM), galectin-3 specific inhibitor G3-C12 (MCE, 30 µM), recombinant galectin-1 protein (Acro, 50 µg/ml), recombinant galectin 3 protein (MCE, 20 µg/ml), recombinant galectin 12 protein (MCE, 10 µg/ml) and galectin 12 antibody (CUSABIO) to the coculture system. The concentration of drug added to the coculture system was selected according to the manufacturer's protocol or published data [21].

### In vitro trogocytosis assay

CD33 purified CAR- T cells stained with CellTrace^™^ Violet (CTV, invitrogen) and GFP^+^ tumor cells were incubated at a ratio of 1:15 at 37 ℃. After 4 and 14 h, the cells were washed and stained and then analyzed by flow cytometry. We measured trogocytosis by CAR, and CTV on the tumor cells or CAR T cells. For trogocytosis rescue tests, 1 µM latrunculin A (Sigma-Aldrich) was added to the coculture system.

### Generation of knockout cell lines

The U937 knockout cell line was generated using the CRISPR/Cas9 technique. CRISPR/Cas9 gene editing was performed by electroporation of the Cas9/gRNA (RNP) complex using the Cell Line Nucleofector Kit C (Lonza). RNP complex containing 100 pmol sgRNA and 10 µg Cas9 protein (Invitrogen), which were pre-mixed for 45 min at room temperature. U937 cells were resuspended with 20 µl RNP transfection buffer and electroporated according to the manufacturer’s protocol in 16-well cuvette strips. Following electroporation, the cells were pipetted. They were cultured in pre-warmed 1640 medium and expanded. Flow cytometry was then used to assess knockdown efficiency after 48 h. We design sgRNAs on the website https://chopchop.cbu.uib.no. The target sequences of the sgRNAs used were as follows: galectin-1:5ʹ—AACCCTCGCTTCAACGCCCACGG-3’, 5ʹ—GTGTGCAACAGCAAGGACGGCGG—3ʹ, 5ʹ—GGTCAGGTTGGCCTGGTCGAAGG—3ʹ, 5ʹ—TCGCCGTGGGCGTTGAAGCGAGG—3ʹ, 5ʹ—CACCATCGTGTGCAACAGCAAGG—3ʹ, 5ʹ—GTGCCTTCGAGTGCGAGGCGAGG—3ʹ; CD33 (Kim et al. [22]), 5ʹ—GTCAGTGACGGTACAGGA—3ʹ [22].

### Xenograft mouse models

NOD.Cg-Prkdc^scid^Il2rg^tm1Wjl^/SzJ (NSG) mice (Beijing Biocytogen) were engrafted with 5 × 10^5^ U937-GFP-Luci cells by i.v. injection to establish tumor models, and 8 × 10^5^ CD33 CAR T cells or CD33 CAR^KR^ T cells were injected i.v. approximately 5 days after tumor inoculation. Tumor burdens were monitored weekly or at indicated time points using the Xenogen IVIS bioluminescence imaging system. The tumor and CAR T cells from the peripheral blood of the mice were analyzed weekly or at indicated time points. The samples were labeled with anti-mouse CD45 and anti-human CD3, CD4 and CD33, CD33 protein with IgG Fc tag antibodies and were analyzed with CantoII (BD Bioscience). Animals were monitored for signs of disease progression and overt toxicity, and survival was analyzed accordingly. All procedures complied with the laboratory animal welfare and ethics committee requirements.

### Statistical analysis

All in vitro experiments were repeated at least three times and statistically analyzed using GraphPad Prism software. Unless otherwise noted, graphs represent either group mean ± SEM values of biological replicates or individual values. The unpaired t-test was used for statistical comparisons in comparing the two groups. Mouse survival curves were compared with the log-rank Mantel-Cox test. When the p-value is less than 0.05, it indicates statistical significance.

## Results

### U937 myeloid leukemia cells induce CD33, CD123 and CLL-1 CAR down-regulation under the conditions of low E: T ratios

**Fig. 1:**
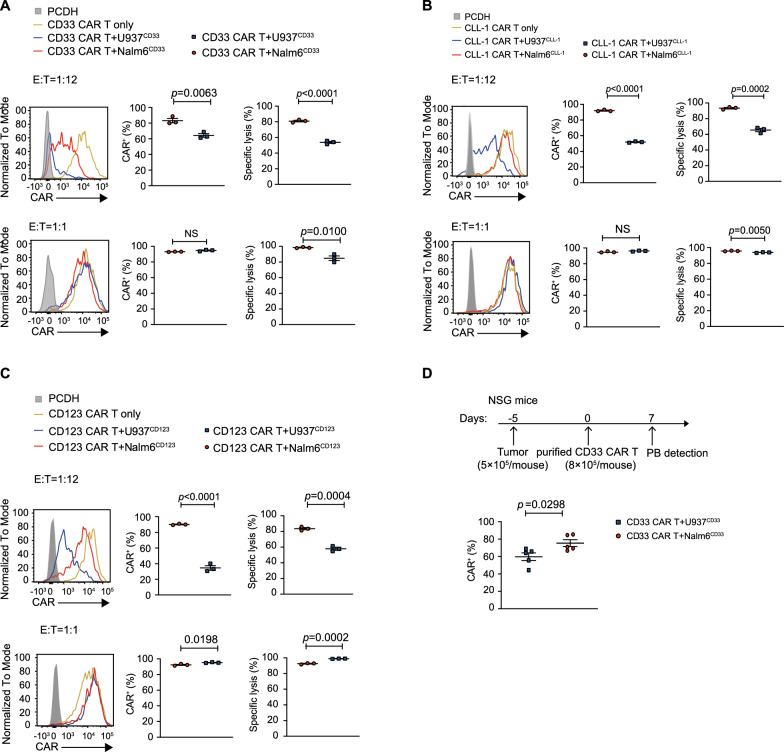
Surface CAR expression is more dramatically down-regulated when exposed to U937 myeloid leukemia cells **A–C**, Surface CD33, CLL-1, and CD123 CAR expression and cytotoxicity of CAR T cells that cocultured with Nalm6^CD33^ or U937^CD33^, Nalm6^CLL−1^ or U937^CLL−1^ cells, Nalm6^CD123^ or U937^CD123^ cells at different effector: target (E: T) ratios. Data shown are mean ± SEM of three experimental replicates. **D**, Surface CAR expression in NSG mouse model. **A**–**D** were representative data from at least three independent experiments with different donors. Assays were performed on day 10 after the initial T-cell culture. Unless otherwise noted, *P* values are determined by unpaired *t*-tests. The numbers on the graphs are *P* values

To investigate whether myeloid leukemia cells directly induce CAR down-regulation, we conducted in vitro and in vivo experiments. Initially, we sorted CAR^+^ cells with a purity greater than 99% and co-cultured them with tumor cells expressing the same level of antigen at various E:T ratios (1:1, and 1:12). As the E:T ratios decreased, the U937^CD33^ group showed a gradual CAR was downregulated by 30%, accompanied by a decline in cytotoxicity. Contrastingly, the Nalm6^CD33^ group maintained an approximate 80% CAR positivity (Fig. 1A). Similarly, at a low E:T ratio of 1:12, U937 myeloid leukemia cell significantly induced CAR down-regulation in CLL-1-targeting or CD123-targeting CAR T cells with decreased cytotoxicity (Fig. 1B–C).

We further observed whether CAR down-regulation occurred in the NSG mice model. Nalm6 and U937 cells expressing the same level of CD33 were injected intravenously into NSG mice, and purified CD33 CAR-T cells were also injected intravenously into these mice five days later. In agreement with the results of the in vitro experiments, the down-regulation of CAR was more pronounced in the U937^CD33^ group than in the Nalm6^CD33^ group (Fig. 1D). These results suggest that under low E:T ratios, U937 myeloid tumor cells induced effective down-regulation of surface CAR, which was observed at the targets CD33, CD123 and CLL-1.

### Several myeloid leukemia cell lines induce CAR down-regulation at a low E: T ratio

**Fig. 2:**
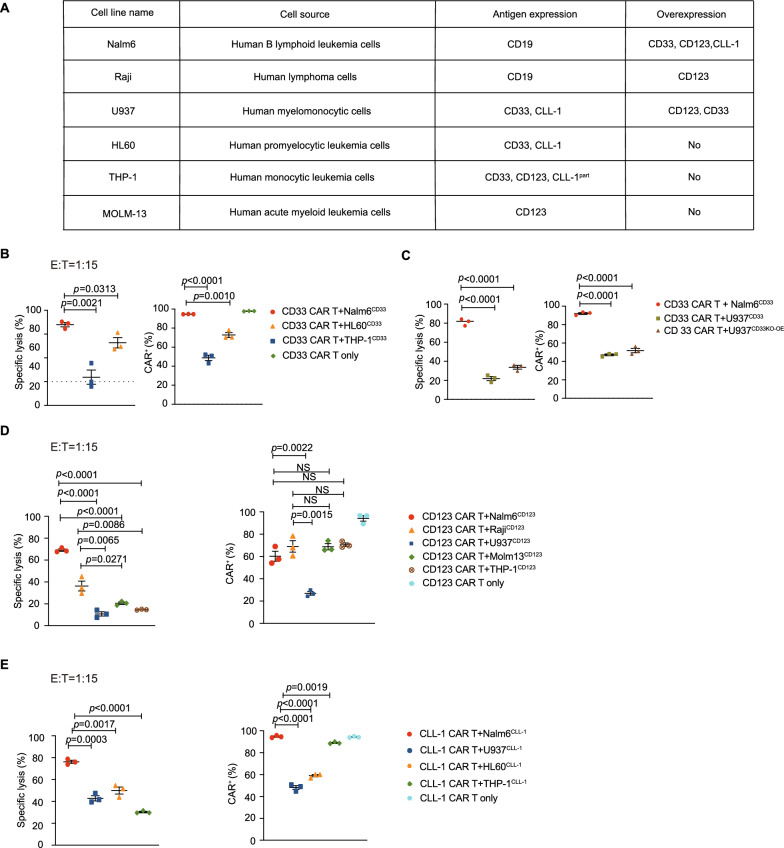
Myeloid leukemia induces CAR down-regulation in different cell lines **A**, Several myeloid and B-lineage tumor cell lines used for in vitro culture are described in terms of their cell source, natural antigen expression and over-expression. **B**, Surface CD33 CAR expression and cytotoxicity of CAR T cells that co-cultured with Nalm6^CD33^, HL60^CD33^ and THP-1^CD33^ at a low E: T ratio of 1:15. **C**, Surface CD33 CAR expression and cytotoxicity of CAR T cells that co-cultured with Nalm6^CD33^, U937^CD33^ and U937^CD33KO−OE^. **D**, Surface CD123 CAR expression of CAR T cells that co-cultured with Nalm6^CD123^, Raji^CD123^, U937^CD123^, MOLM-13^CD123^ and THP-1^CD123^ at a low E:T ratio of 1:15. Cytotoxicity of CD123 CAR T cells that co-cultured with Nalm6^CD123^, Raji^CD123^, U937^CD123^, MOLM-13^CD123^ and THP-1^CD123^. **E**, Surface CLL-1 CAR expression and cytotoxicity of CAR T cells co-cultured with Nalm6^CLL−1^, U937^CLL−1^, HL60^CLL−1^ and THP-1^CLL−1^ at a low E: T ratio of 1:15. **B–E**, were representative data from at least three independent experiments. Assays were performed on day 10 after the initial T-cell culture. Unless otherwise noted, *P* values are determined by unpaired *t*-tests. The numbers on the graphs are *P* values

To determine if the down-regulation of CD33, CD123, CLL-1 CAR applies to different myeloid leukemia cell lines other than U937, we also conducted the co-cultured experience using THP-1, MOLM-13 and HL60 myeloid leukemia cell lines with CAR T cells. The cellular origin and the antigenic expression of the different tumor cell lines that used in our experiments were summarized in the table (Fig. 2A). With low 1:15 E:T ratio, CD33 CAR T cells co-cultured with myeloid leukemia cells, HL60^CD33^ and THP-1^CD33^, showed poorer anti-tumor capacity compared to Nalm6^CD33^. Correspondingly, HL60^CD33^ and THP-1^CD33^ also significantly induced the down-regulation of CAR (Fig. 2B). We co-cultured CD33 CAR T cells with the U937 cell line, which naturally expresses and over-expresses the CD33 antigen, and both significantly induced CAR down-regulation and reduced the killing capacity of CAR T cells (Fig. 2C).

Then, we cocultured CD123 CAR T cells with Nalm6^CD123^, Raji^CD123^, U937^CD123^_,_ MOLM-13^CD123^ and THP-1^CD123^ with low 1:15 E:T ratio. CD123 CAR-T cells cocultured with U937^CD123^, MOLM-13^CD123^ and THP-1^CD123^ myeloid tumor cells had poor antitumor activity compared to coculturing with B-lineage tumor cells Nalm6^CD123^ and Raji^CD123^, and U937^CD123^ significantly induced CAR down-regulation. However, the MOLM-13^CD123^ and THP-1^CD123^ myeloid tumor cell lines did not induce down-regulation of CAR compared to B-lineage tumor cell lines Nalm6^CD123^ and Raji^CD123^. (Fig. 2D). Additionally, with low 1:15 E:T ratio, we co-cultured CLL-1 CAR T cells with U937^CLL−1^, HL60^CLL−1^ and THP-1^CLL−1^ had poor antitumor activity than Nalm6^CLL−1^, and U937^CLL−1^, HL60^CLL−1^, THP-1^CLL−1^ induced CAR down-regulation (Fig. 2E).

### CAR down-regulation is due to lysosomal degradation of CAR after internalization, not trogocytosis

**Fig. 3:**
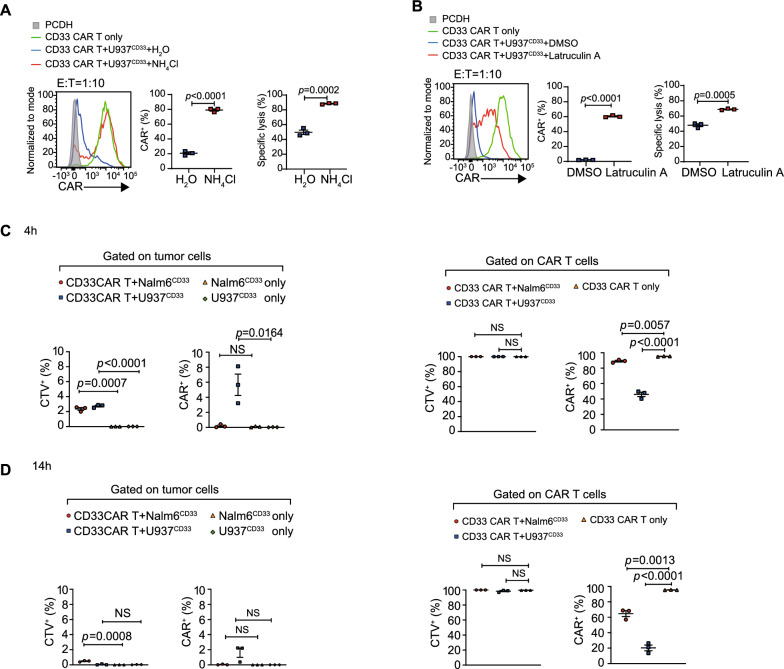
CAR down-regulation is due to lysosomal degradation of CAR **A**, **B**, Surface CAR expression and cytotoxicity of CAR T cells that cocultured with U937^CD33^ cells for 12 h in the presence of 20 mM NH_4_Cl (**A**) or 1 µM latruculin A (**B**), at a 1:10 E:T ratio. Data shown are mean ± SEM of three experimental replicates. **C**, The percentage of CTV^+^, CAR^+^ cells in tumor cells cocultured with CD33 CAR T cells or tumor cells only and the CTV^+^, CAR^+^ cell percentage in CD33 CAR T cells after coculture with U937^CD33^ or Nalm6^CD33^ cells or CAR-T cells only for 4 h at a 1:15 E:T ratio. Data shown are mean ± SEM of three experimental replicates. **D**, The percentage of CTV^+^, CAR^+^ cells in tumor cells cocultured with CD33 CAR T cells or tumor cells only and the CTV^+^, CAR^+^ cell percentage in CD33 CAR T cells after coculture with U937^CD33^ or Nalm6^CD33^ cells or CAR-T cells only for 14 h at a 1:15 E:T ratio. Data shown are mean ± SEM of three experimental replicates. **A**–**D**, were representative data from at least three independent experiments. Assays were performed on day 10 after the initial T-cell culture. Unless otherwise noted, *P* values are determined by unpaired *t*-tests. The numbers on the graphs are *P* values

In activated T cells, lysosomes play a vital role in the degradation of the CD3 complex [23]. We found that NH_4_Cl, an inhibitor of lysosomal degradation, was added to the co-culture system and can effectively mitigate CAR downregulation (Fig. 3A). Furthermore, latrunculin A, an inhibitor of actin polymerization, can also alleviate CAR downregulation when added to the co-culture system (Fig. 3B). Both NH_4_Cl and latrunculin A notably restored CAR T cell-mediated cytotoxicity against U937^CD33^ cells (Fig. 3A, B). In a study by Hamieh et al. impaired CAR T-cell function was associated with antigen trogocytosis [16]. However, in our study, the increased CAR down-regulation was not a consequence of enhanced trogocytosis. This is supported by the evidence that there was no increase in cell membrane or CAR molecule exchange between CTV^+^CAR T cells and tumor cells from 4 to 14 h, accompanied by increased down-regulation of CAR. (Fig. 3C, D).

### Galectin-1 is highly expressed in myeloid leukemia cells

**Fig. 4:**
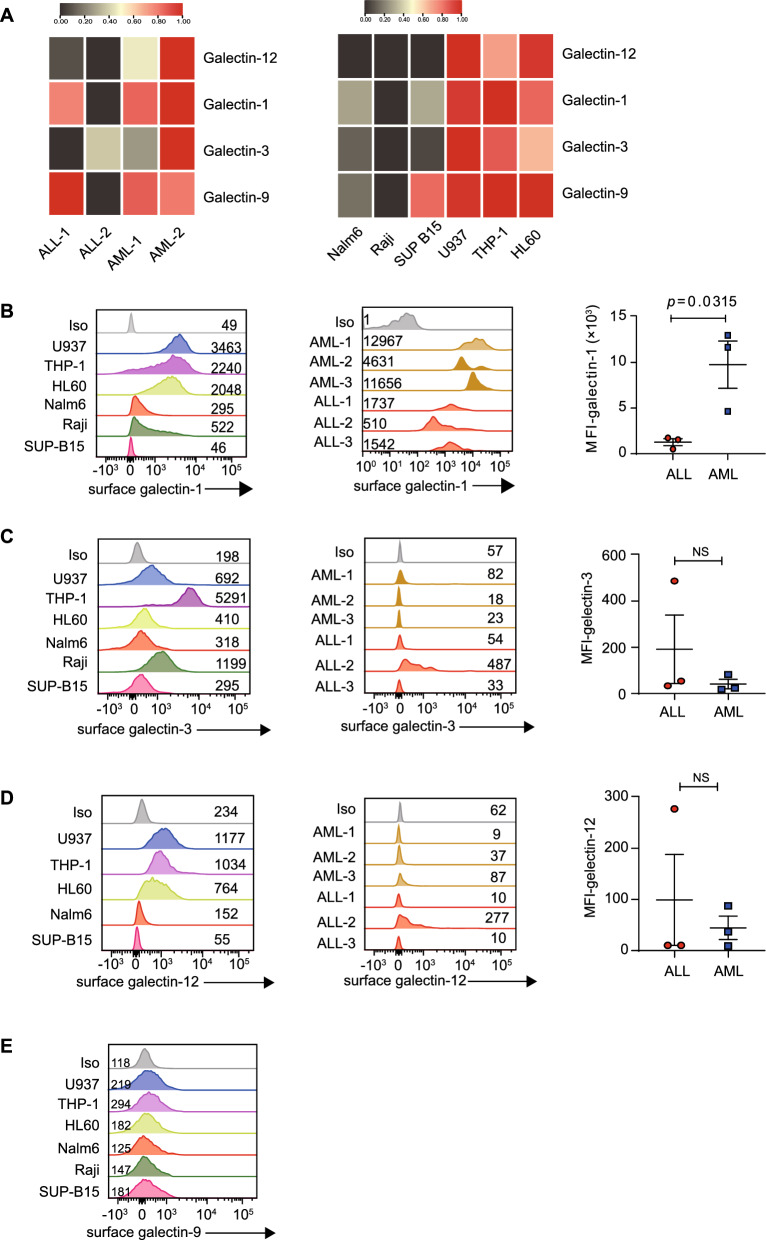
Galectins are highly expressed in myeloid leukemia cells **A**, Heat maps showing suppressive genes differentially expressed between myeloid and lymphoid leukemia cell lines and between primary AML and ALL samples. Genes are those that are up or down-regulated more than two-fold, except galectin-9 in primary AML and ALL samples. AML and ALL primary samples had two biological replicates, respectively. **B–E**, Histograms showing the expression of galectin-1, galectin-3, galectin-12, and galectin-9 in AML and ALL cell lines; Histograms and the MFI of galectin-1, galectin-3,and galectin-12 in primary AML and ALL samples. Data shown are mean ± SEM of three experimental replicates

Next, our focus shifted to identifying the mediators responsible for CAR T-cell down-regulation in myeloid leukemia. We performed transcriptome sequencing on various AML and ALL cell lines and primary samples to gain further insights. The analysis revealed elevated expression of transcripts encoding inhibitory molecules, including *galectin-1, 3, 9, and 12* in AML cell lines or primary samples (Fig. 4A). Myeloid leukemia cell lines consistently exhibited higher galectin-1 and 12 protein levels than lymphoid leukemia cell lines. Among them, the U937 cell line had the highest expression levels of galectin-1 protein. The highest expression of galectin-3 was found in the myeloid cell line THP-1. In the U937 and HL60 myeloid leukemia cell lines, galectin-3 expression was observed to be higher compared to the lymphoid leukemia cell lines Nalm6 and SUP-B15, but lower than in Raji. (Fig. 4A–D). However, the expression of galectin-9 protein did not show significant differences (Fig. 4E).

Furthermore, primary AML samples demonstrated significantly higher expression of galectin-1 proteins compared to ALL samples (Fig. 4B). In contrast, galectin-3 and galectin-12 proteins did not show differential expression between AML and ALL primary samples (Fig. 4C–D). These findings shed light on the potential involvement of galectin-1 in mediating the CAR T-cell down-regulation in myeloid leukemia.

### Myeloid leukemia cells use galectin-1 to block CAR T cell function

**Fig. 5:**
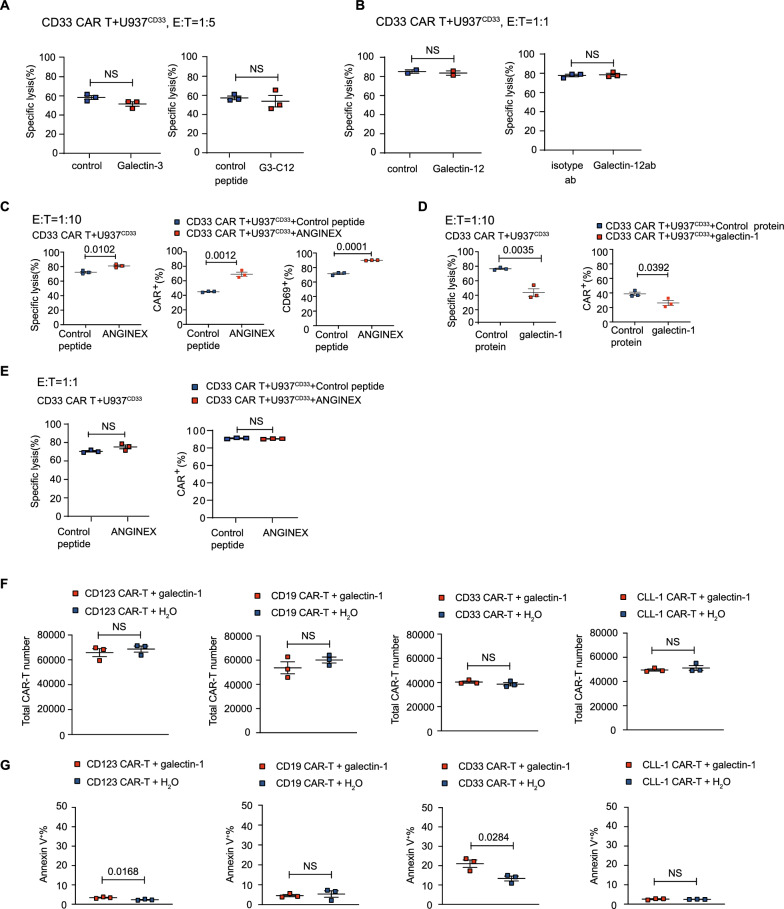
Myeloid leukemia cells use galectin-1 to block CAR T cells function **A**, The specific lysis after coculture with U937^CD33^ cells at a 1:5 in the presence of recombinant galectin 3 protein (20 µg/ml) and galectin-3 specific inhibitor G3-C12 (30 µM). **B**, The specific lysis after coculture with U937^CD33^ cells at a 1:1 in the presence of recombinant galectin 12 protein (10 µg/ml) and galectin 12 antibody. **C**, The specific lysis, the percentage of CD69 expression in CD33 CAR T cells, and the percentage of surface CAR expression in CD33 CAR T cells, after coculture with U937^CD33^ cells at a 1:10 E:T ratio for 12 h, in the presence of 10 µM galectin-1 inhibitory peptide (ANGINEX). **D**, The specific lysis and the percentage of surface CAR expression in CD33 CAR T cells, after coculture with U937^CD33^ cells at a 50 µg/ml recombinant galectin-1 protein. **E**, The specific lysis and the percentage of surface CAR expression in CD33 CAR T cells, after coculture with U937^CD33^ cells at a 1:1 ratio for 12 h in the presence of 10 µM galectin-1 inhibitory peptide (ANGINEX). F, Total number of CD123, CD33,CD19 and CLL-1 CAR-T cells in the experimental group with 50 µg/ml recombinant galectin-1 protein and in the control group. **G**, Annexin V positivity in CD123, CD33, CD19 and CLL1 CAR-T cells from experimental and control groups using 50 µg/ml recombinant galectin-1 protein

Neither the recombinant galectin-3 nor galectin-12 proteins, the galectin-3-specific inhibitory peptide (G3-C12), or the galectin-12-specific antibody showed significant effects on the killing capacity of CAR T cells (Fig. 5A–B). However, in our cytotoxicity assay at an E:T ratio of 1:10, the galectin-1-specific inhibitory peptide (anginex) successfully rescued the killing defect and T-cell activation (Fig. 5C), whereas recombinant galectin-1 protein added to the co-culture system inhibited the killing activity (Fig. 5D). Furthermore, we observed that anginex upregulated the expression of CAR (Fig. 5C), while the recombinant galectin-1 protein added to the coculture system downregulated it (Fig. 5D). It is worth noting that, with a higher E:T ratio, anginex did not affect the killing function or the expression of CAR in CAR T cells (Fig. 5E). We also attempted to knock out galectin-1 in U937^CD33^, but unfortunately, our attempts were unsuccessful.

To test the effect of galectin-1 protein on T-cell apoptosis, we added 50 µg/ml of galectin-1 protein to purified CD33, CD19, CD123 and CLL-1 CAR-T cells and found that the total number of viable CAR-T cells in the experimental group did not decrease significantly compared to the control group (Fig. 5F). There was no increase in Annexin V-positive cells in CD19 and CLL-1 CAR-T cells, while a slight increase in Annexin V-positive cells in CD123 and CD33 CAR-T cells after the addition of galectin-1 protein (Fig. 5G).

### CD33 CAR^KR^ T cells are effective in vitro and less effective in vivo

**Fig. 6:**
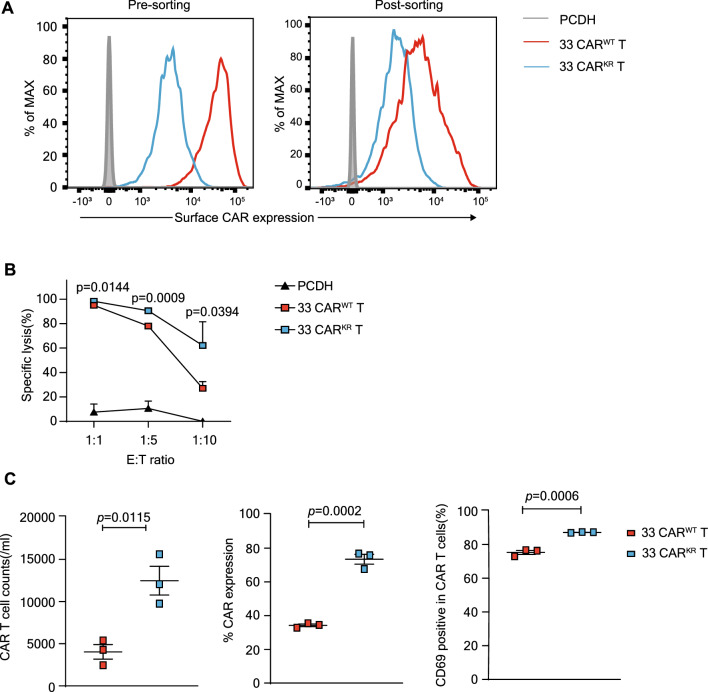
CD33 CAR^KR^ T cells are effective in vitro **A**, the CAR expression before and after cell sorting. **B**, cytotoxicity of CD33 CAR T cells, CD33^KR^ CAR T cells, and PCDH CAR T cells that co-cultured with U937^CD33^ cells at different effector: target (E:T) ratios. **C,** CAR T cell number, the CAR expression, and CD69 positivity expression in CD33 CAR T cells and CD33^KR^ CAR T cells. **A–C**, were representative data from at least three independent experiments. Assays were performed on day 10 after the initial T-cell culture. Unless otherwise noted, *P* values are determined by unpaired *t*-tests. The numbers on the graphs are *P* values

**Fig. 7:**
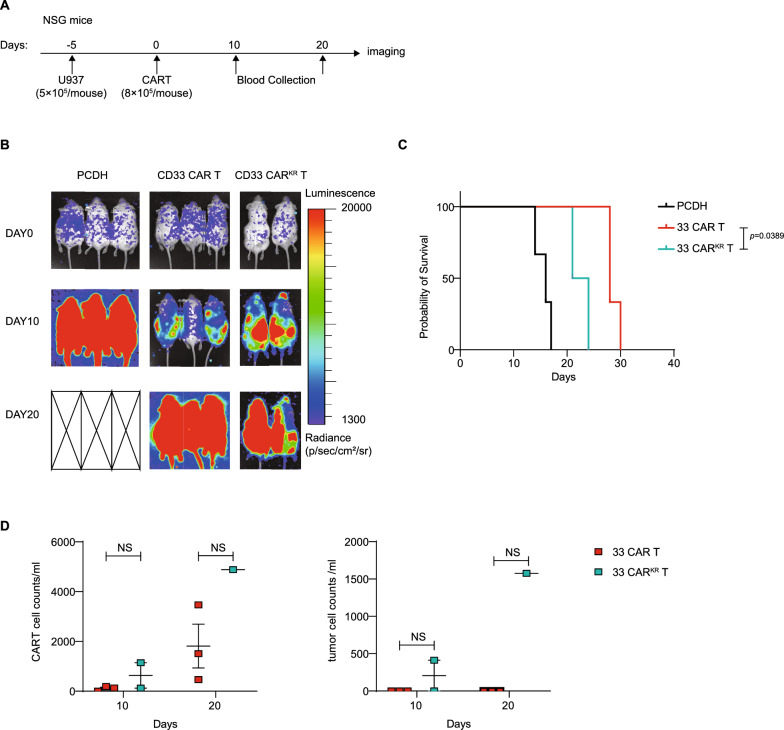
CD33 CAR^KR^ T cells are less effective in vivo **A**, Schematic of the acute myeloid leukemia model. **B**, Representative bioluminescence images of tumor growth. **C,** Kaplan-Meier curve showing the survival of mice in each experiment group. *P* value was determined by the log-rank Mantel-Cox test. **D**, CAR T cell and tumor cell counts in peripheral blood 10 and 20 days after CAR T infusion. Each dot represents one mouse, n = 2 or 3 mice per group

Li et al. generated a new CAR by switching all cytoplasmic lysines to arginines to prevent CAR downregulation (CAR^KR^ [35]. To specify the impact of CAR down-regulation on cytolysis, we generated CD33 CAR^KR^ using the same method. The sorted and purified cell experiments were performed to match the expression levels of the two cell lines to ensure better comparability. (Fig. 6A). CD33 CAR^KR^ T cells killed better than CAR^WT^ cells, especially at low E:T ratios (1:5 and 1:10), but at a high E:T ratio of 1:1, the killing advantage is not particularly obvious (Fig. 6B). CD33 CAR^KR^ T cells upregulated CAR and CD69 expression and increased CAR T cell counts (Fig. 6C). Accordingly, we speculated that CAR down-regulation may impair CAR T-cell killing in AML. We also observed the effect of CAR^KR^ in vivo (Fig. 7A) and found that the CAR^KR^ group was not able to limit tumor progression (Fig. 7B) while having a worse survival (Fig. 7C) compared to the CAR^WT^ group. We found that the number of CAR T cells and tumor cells in NSG mice of CD33 CAR^KR^ group was higher than the CD33 CAR group (Fig. 7D). The above data suggests that the CD33 CAR^KR^ T may be less effective in vivo. Unfortunately, one mouse in the CD33 CAR^KR^ group died due to improper experimental manipulation, leaving only 2 mice. As there was no significant effect in the in vivo experiments, independent replications were not performed.

### Galectin-1-specific inhibitory peptide is less effective in vivo

**Fig. 8:**
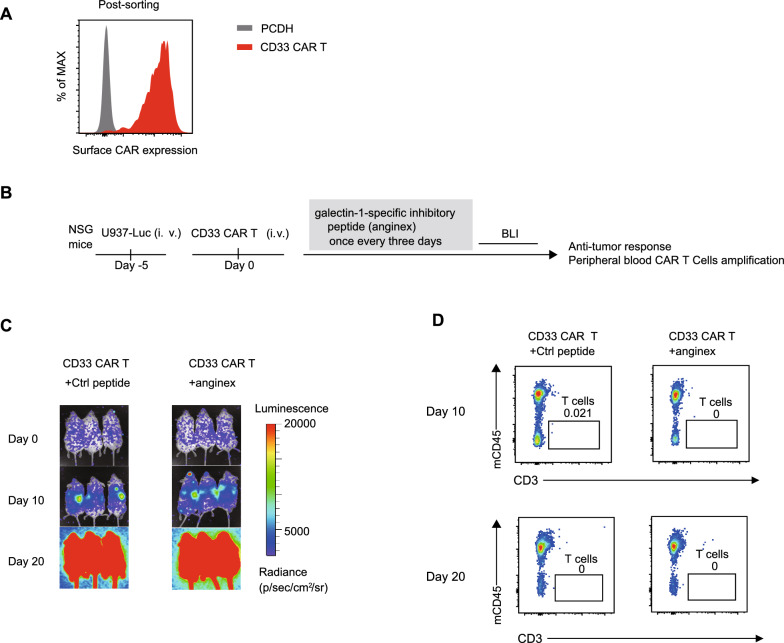
Galectin-1-specific inhibitory peptide is ineffective in vivo **A**, The CD33 CAR T cells was sorted and purified by FACS. **B**, Schematic of the acute myeloid leukemia model. **C**, Representative bioluminescence images of tumor growth. **D**, T cells in peripheral blood 10 and 20 days after CAR T infusion. Each dot represents one mouse, n = 3 mice per group

To further confirm this potential of the galectin-1-specific inhibitory peptide (anginex) in vivo, NSG mice established with 5 × 10^5^ U937 tumor cells received a single dose of 8 × 10^5^ CD33 CAR T cells followed by intraperitoneal injection of the anginex and control peptide (10 mg/kg (body weight) every 3 days) (Fig. 8B). The CD33 CAR T cells were sorted and purified by FACS (Fig. 8A). The anginex-treated NSG mice did not show better anti-tumor effects and progressed just as fast compared to control mice (Fig. 8C). The peripheral blood of the mice was examined by flow cytometry and it was found that anginex-treated mice, like control mice, did not achieve expansion of T cells (Fig. 8D). These results suggest that galectin-1-specific inhibitory peptides may need to be combined with other drugs to exert significant antitumour efficacy in vivo.

## Discussion

CAR T-cell therapy has not achieved satisfactory efficacy in treating AML. At the same time, the underlying molecular mechanisms remain largely unknown [24–29]. This study revealed a previously unrecognized mechanism by which AML resists CAR T-cell therapy. We found a potential mechanism that AML cells can inhibit CAR T cells’ anti-tumor function, and this effect is galectin-1-dependent blockade of surface CAR expression. While CAR down-regulation has been previously linked to exposure to target antigens and limited CAR T-cell activity in ALL [35], the factors contributing to the extent of CAR down-regulation were not well defined. We showed that CAR down-regulation was more significant in the AML than in the ALL microenvironment. CAR down-regulation was more pronounced at low E:T ratios in vitro and in vivo experiments. The U937 cell line, which naturally expresses CD33 and CLL-1, is widely used for in vitro experiments and in vivo mouse models in preclinical AML studies [30–32]. The Nalm6 cell line, which naturally expresses CD19 and CD22, is also widely used for in vitro experiments and in vivo mouse models in preclinical studies of B-cell malignancies [33, 34]. Therefore, we first selected two representative cell lines, U937 and Nalm6, for in vitro experiments to validate CAR down-regulation in the myeloid tumor microenvironment. To further investigate whether this phenomenon applies to other myeloid and B-lineage cell lines, We selected several commonly used myeloid leukemia cell lines, MOLM-13, HL60, THP-1, and the B-lineage derived cell line Raji. When CD33 and CLL-1 CAR T cells were co-cultured with different myeloid tumor cells such as U937, THP-1 and HL60, all of them induced CAR down-regulation, however, THP-1 and MOLM-13 cell lines did not induce CAR down-regulation when they were co-cultured with CD123 CAR T cells. These results suggest that CAR down-regulation induced by the myeloid tumor microenvironment may not be fully applicable to target CD123. Furthermore, some of the tumor cell lines used in the in vitro experiments over-expressed antigens, while others naturally expressed antigens. To verify the impact of natural expression and overexpression of antigens on inducing CAR down-regulation in myeloid tumor cell lines, we conducted a co-culture assay using the U937 cell line, which naturally expresses the CD33 antigen and overexpresses it again after CD33 knockdown, with CD33 CAR T cells and found that both induced CAR down-regulation and the degree of down-regulation was basically the same. Considering that the intensity of antigen expression may also have an impact on inducing CAR downregulation, we sorted tumor cells with consistent levels of CD33, CD123 and CLL-1 antigen expression for co-culture with CAR T cells.

We found the CAR down-regulation was mainly dependent on internalization and lysosomal activity but not on trogocytosis between CAR T and tumor cells. Li et al. reported that CAR ubiquitination regulates the down-regulation of CAR by preventing internalized CAR and promoting their lysosomal degradation. CAR ubiquitination can be prevented by replacing all cytoplasmic lysines with arginine. They also verified that CD19 CAR^KR^ is effective *in vivo* [35]. Based on this, we designed CD33 CAR^KR^ to avoid CAR ubiquitination, which enhanced the activation and anti-tumor activity of CAR-T cells in vitro experiments. However, in the NSG mouse model, the therapeutic efficacy of CD33 CAR^KR^-T cells was worse than that of CD33 CAR^WT^-T cells. Several factors may be responsible for the ineffectiveness of CD33 CAR^KR^ T cells in vivo. First, the tumor microenvironment in vivo is complex, such as interactions between different cell types, cytokines, and signaling molecules, which may affect CAR-T cell responses differently than simplified in vitro coculture conditions. In addition, although we sorted CAR-T cells with identical CAR expression, the MFI of CAR in CD33 CAR-T cells decreased over time, which may be related to complexity, including interactions with tumor cells, immune cells, cytokines, and the tumor microenvironment, all of which affect the stability of CAR. Reduced MFI expression in CD33 CAR^KR^ T cells may lead to weakened interactions between CAR T cells and tumor cells, which in turn may affect the ability of CAR T cells to recognize and eliminate target cells effectively. Furthermore, reduced MFI may affect downstream signaling pathways, affecting function of CAR T cells and reducing their therapeutic potential.

Notably, our study identifies galectin-1, which is highly expressed in AML cell lines and AML primary samples, as a crucial factor in inducing CAR down-regulation and hindering the cytotoxicity of CAR T cells. Although previous studies have suggested a potential role of galectins in modulating lysosomal activity, the precise mechanism of how galectin-1 affects CAR expression needs to be further investigated. Galectin-1 could exert some inhibitory effects on CAR T cells independent of CAR down-regulation. Previous studies have suggested that galectin-1 can impede antigen binding to the TCR and induce T-cell apoptosis [36, 37]. We did not observe a significant effect of galectin-1 on the survival of CAR T cells. The addition of galectin-1 protein did not reduce the number of viable CAR T cells in the in vitro. However, galectin-1 protein slightly induced apoptosis in CAR T cells, and the number of Annexin V^+^ cells in CD33- and CD123- CAR T cells was slightly increased by the addition of galectin-1 protein compared to the control group. To further investigate the function of galectin-1 in CAR down-regulation, we performed knockdown experiments, designed six sgRNAs on different exons of the galectin-1 gene on the website https://chopchop.cbu.uib.no, tried to knockdown the galectin-1 gene in U937 cells, and then performed related mechanism studies. Unfortunately, none of the six sgRNAs had any effect. We also designed siRNAs targeting the galectin-1 gene, but these were also ineffective. This suggests that the galectin-1 gene may be difficult to knock out. In conclusion, further analysis is warranted to determine whether galectin-1 interferes with the target antigen and CAR interaction. We performed in vivo experiments to further validate the effect of the galectin-1 inhibitory peptide. As this was an exploratory experiment, we followed the design of the in vitro experiments and used three mice for the experimental and control groups, but found that neither the anti-tumor effect nor the T-cell expansion showed an advantage. Therefore, we did not expand the sample size of mice for further study. The lower efficacy of galectin-1 inhibitory peptide in vivo may be due to several factors. First, galectin-1 inhibitory peptide partially rescued CAR down-regulation in vitro experiments, but did not fully restore CAR expression, this suggests that there may be other mechanisms besides galectin-1 that induce CAR down-regulation. Secondly, galectin-1 inhibitory peptide may not be the most potent inhibitor of galectin-1 protein expression. Finally, Galectin-1 inhibitory peptides injected intraperitoneally into mice may be subject to pharmacokinetic and microenvironmental effects when acting in vivo. In conclusion, we need to continue to explore other factors that induce CAR down-regulation, as well as drugs that can provide stronger inhibition of the galectin-1 protein, and consider combining multiple drugs to rescue CAR down-regulation and thus enhance the antitumor effects of CAR T cells.

This study has several limitations. First, although our research has identified galectin-1 as a mediator of AML resistance to CAR T-cell therapy, the signaling pathways downstream of galectin-1 need to be further studied. In addition, the exact mechanisms of CAR down-regulation in AML remains to be further explored and validated. Ultimately, neither the CD33^KR^ CAR nor the galectin-1 inhibitory peptide were very effective in in vivo experiments, this may be due to the complex microenvironment in mice. It may be necessary to consider using several drugs in combination to achieve better therapeutic outcomes. In the case of the mouse study, we are considering additional assays at the protein and RNA levels next to further support the findings.

In conclusion, we identified mechanisms by which AML resists CAR T-cell therapy and proposed a strategy to overcome it. Our study provides the first evidence supporting that myeloid leukemia-derived galectin-1 down-regulates CAR expression to hinder the cytotoxicity of CAR T cells. Our findings offer necessary information for the future design of targeted cellular therapies for AML.

### Supplementary Information

**Additional file 1: Data S1.** Information of antibodies.

## Data Availability

All requests for raw and analyzed data will be subject to review by the corresponding authors to determine whether there are any intellectual property or confidentiality considerations.
